# Analysis of cumulative live birth rate outcomes of three ovarian stimulation protocols in patients after laparoscopic cystectomy of ovarial endometrioma: a retrospective cohort study

**DOI:** 10.1186/s12978-023-01671-3

**Published:** 2023-08-29

**Authors:** Jiaheng Li, Yijiang Li, Mengnuo Li, Xianling Zhao, Wei Zheng, Junwei Zhang, Yuchao Zhang, Jing Li, Yichun Guan

**Affiliations:** Reproduction Center, The Third Affiliated Hospital of ZhengZhou University, Zhengzhou, Henan China

**Keywords:** Diminished ovarian reserve, Ovarial endometrioma, Cumulative live birth rate

## Abstract

**Background:**

Previous studies have reported that after laparoscopic cystectomy of ovarial endometrioma, the ovarian response to gonadotropin (Gn) significantly decreased. However, for patients with diminished ovarian reserve (DOR) after ovarian surgery, how to choose the most appropriate controlled ovarian hyperstimulation protocol has not been concluded. Compared with the traditional agonist regimen, the gonadotropin (Gn)-releasing hormone (GnRH) antagonist, microstimulation, and progestin-primed ovarian stimulation (PPOS) protocols are simple to operate and have a shorter cycle, which are often used in patients with DOR. So the purpose of our study is to compare the assisted reproductive outcomes of these three controlled ovarian hyperstimulation protocols in patients with DOR following laparoscopic cystectomy of ovarial endometrioma.

**Methods:**

In this retrospective cohort study, 89 patients with DOR who had undergone in vitro fertilisation/intracytoplasmic sperm injection at the Department of Reproductive Medicine at the Third Affiliated Hospital of Zhengzhou University from 1 to 2018 to 31 December 2020 were included. According to the controlled ovarian hyperstimulation protocols employed, the patients were divided into GnRH antagonist (38 patients), PPOS (27 patients), and microstimulation (24 patients) groups. The basic data and clinical outcomes of the three groups were compared. The main outcome measure was the cumulative live birth rate.

**Results:**

No significant differences in the age of the female patients and their spouses and female patients’ body mass index and basal endocrine levels (follicle-stimulating hormone and oestradiol) were noted among the three groups (P > 0.05). The GnRH antagonist group had higher antral follicle counts, greater endometrial thickness on the human chorionic Gn injection day, greater number of oocytes retrieved, and higher two pronuclear embryo counts than did the other two groups. However, the starting dosage of Gn was lower in the GnRH antagonist group than in the other two groups. The microstimulation group had a significantly higher oocyte output rate and high-quality embryo rate than did the other two groups (P < 0.05). No significant differences in the total dosage of Gn, cumulative pregnancy rate, cumulative live birth rate, viable embryo rate, and blastocyst formation rate were observed among the three groups (P > 0.05).

**Conclusion:**

In conclusion, for patients aged under 40 years who experienced DOR after laparoscopic cystectomy of ovarial endometrioma, GnRH antagonist protocol and PPOS protocol can obtain better ovulation induction outcomes and cumulative live birth rate than microstimulation protocol, and are more suitable ovulation induction protocols.

## Introduction

Endometriosis is a common oestrogen-dependent gynaecological disease. Ovarian endometriotic cysts, the most common manifestation of endometriosis, are caused by the recurrent bleeding of the ectopic endometrial tissue in the ovaries during menstruation. This phenomenon results in the formation of ectopic cysts that are filled with accumulated old blood and are also known as chocolate cysts. Laparoscopic cystectomy of ovarial endometrioma is currently the first-line treatment for infertility caused by these cysts [1]. This surgical procedure can not only eliminate ectopic lesions but also restore the normal pelvic anatomy, thus alleviating symptoms and improving women’s quality of life. However, this treatment can damage the normal ovarian tissue, resulting in diminished ovarian reserve (DOR) [2]. Studies have reported that after laparoscopic cystectomy of ovarial endometrioma, the ovarian response to gonadotropin (Gn) significantly decreased [3], with the incidence of ovarian failure ranging from 2.4–13% [4].Controlled ovarian hyperstimulation (COH) is one of the key steps in assisted reproductive technology (ART), These patients may have a low number of developing follicles, a high dose of Gn, a high cycle cancellation rate, a low number of oocytes retrieved and available embryos, which may lead to low pregnancy rate and live birth rate. It is very important to develop individualized COH protocol according to different populations for assisted reproductive outcomes, especially for patients with DOR after laparoscopic cystectomy of ovarial endometrioma. It is of great significance to develop standardized COH protocol to improve the cumulative live birth rate (CLBR). Gonadotropin (Gn)-releasing hormone (GnRH) antagonist, microstimulation, and progestin-primed ovarian stimulation (PPOS) protocols are commonly used for ovarian stimulation in patients with DOR, but there is no study to compare the assisted reproductive outcomes of these three protocols in patients with DOR after laparoscopic cystectomy of ovarial endometrioma. Therefore, this study retrospectively analysed the effects of these three protocols on CLBR to determine a more suitable ovulation induction regimen for patients experiencing DOR following laparoscopic enucleation of ovarian cysts. The findings of this study provide valuable guidance for the clinical treatment of such patients.

## Materials and methods

### Patients

In this retrospective study, we included patients who had received in vitro fertilisation (IVF) treatment or intracytoplasmic sperm injection (ICSI) at the Department of Reproductive Medicine at the Third Affiliated Hospital of Zhengzhou University between 1 and 2018 and 31 December 2020 and had undergone a single laparoscopic cystectomy of ovarial endometrioma. Because the goal was to compare the effectiveness of different controlled ovarian hyperstimulation protocols, patients were divided into three groups: a Gn-releasing hormone (GnRH) antagonist group, a microstimulation group, and a progestin-primed ovarian stimulation (PPOS) group.

### Inclusion criteria

The inclusion criteria were as follows: (1) age ≤ 40 years; (2) DOR [5], evidenced by the presence of either (a) an anti-Mullerian hormone (AMH) level of < 1.1 ng/mL, (b) an antral follicle count (AFC) of < 5–7 in both the ovaries, or (c) a basal follicle-stimulating hormone (FSH) level of ≥ 10 IU/L in two consecutive menstrual cycles; (3) a history of a single laparoscopic cystectomy of ovarial endometrioma; and (4) first IVF/ICSI-assisted pregnancy cycle.

### Exclusion criteria

The exclusion criteria were as follows: (1) endocrine-related diseases, such as polycystic ovary syndrome and hyperprolactinaemia; (2) any chromosomal abnormality in either spouse; (3) uterine malformations; (4) a history of recurrent miscarriage; (5) diagnosis of adenomyosis through surgery, ultrasound, or magnetic resonance imaging; (6) cycles with incomplete data; and (7) cycles involving the preimplantation genetic diagnosis and preimplantation genetic screening.

### Controlled ovarian hyperstimulation protocols

1) GnRH antagonist protocol: Based on the patient’s age, body mass index (BMI), and ovarian reserve, ovulation induction was initiated between the second and fourth day of menstruation by administering Gn. The primary agents used were urinary Gn (Zhuhai Lizhu Group, Lizhu Pharmaceutical Factory) and recombinant FSH (Konafen, Merck, Germany), Procon (MSD, USA), or Lishenbao (Zhuhai Lizhu Group, Lizhu Pharmaceutical Factory). Upon reaching an average follicular diameter of 11 to 12 mm and a serum oestradiol level of > 500 ng/L, the patients were administered GnRH antagonists (Citrek, Merck Serrano, Switzerland) at a dose of 0.25 mg/d.

2) Microstimulation protocol: Between the second and fourth day of menstruation, patients were orally administered 2.5 mg/d of letrozole (Jiangsu Hengrui Pharmaceutical Co., Ltd.) or 50 mg/d of clomiphene citrate (Shanghai Hengshan Pharmaceutical Co., Ltd.). Simultaneously, they were administered an intramuscular injection of human menopausal Gn (urotropin for injection, Zhuhai Lizhu Group, Lizhu Pharmaceutical Factory) at an initial dose of 150 IU/d, which was continued until the human chorionic Gn (hCG) injection day.

3) PPOS: From the second to fourth day of menstruation, patients were orally administered 6–10 mg/d of medroxyprogesterone acetate (Zhejiang Xianju Pharmaceutical Co., Ltd.) and injected 150–225 U/d of Gn until the hCG injection day.

The Gn dosage was maintained or adjusted during treatment based on follicle growth and serum hormone levels. When at least one dominant follicle reached a diameter of ≥ 20 mm or three follicles reached a diameter of ≥ 18 mm, hCG (Zhuhai Lizhu Group, Lizhu Pharmaceutical Factory), recombinant hCG (Aize, Merck Serrano, Switzerland), or Dafirin (Iproxen, France) were administered to trigger ovulation. Egg retrieval was performed after 36 h under vaginal ultrasound guidance.

### Embryo transfer

In the GnRH antagonist protocol, given the absence of any contraindications, a fresh cycle transfer was performed first depending on the endometrial thickness and serum hormone levels. Two fresh cleavage-stage embryos or one blastocyst was transferred on day 3 or 5 after oocyte retrieval. If pregnancy was not achieved, frozen–thawed embryo transfer (FET) was performed in subsequent cycles. In the microstimulation and PPOS protocols, vitrification freezing technology was employed for total embryo freezing. Different FET plans were developed based on the specific situation of each patient. Endometrial development or follicle growth and serum hormone levels were continuously monitored, and endometrial preparation was timed accurately. Two frozen–thawed cleavage-stage embryos were transferred 3 days after endometrial preparation or one frozen–thawed blastocyst was transferred 5 days after endometrial preparation.

### Pregnancy diagnosis

Blood hCG levels were measured 14 days after transplantation. Clinical pregnancy was considered if an ultrasound examination performed 30 days after transplantation revealed a gestational sac.

### Observation indicators

The primary outcome was the cumulative live birth rate. In an IVF/ICSI cycle (which includes one oocyte retrieval cycle, fresh embryo transfer, and subsequent FETs), the number of cycles resulting in the first live birth (defined as ≥ 28 weeks of gestation) was used as the numerator and the number of oocyte retrieval cycles was used as the denominator. The observation was continued until one live birth was observed or all embryos were utilised [6, 7].

The secondary outcomes were patient general characteristics, the number of oocytes retrieved, the oocyte output rate, the number of two pronuclear (2PN) embryos, the viable embryo rate, the high-quality embryo rate, the blastocyst formation rate, and the cumulative pregnancy rate.

### Statistical analysis

All statistical analyses were performed using SPSS (Statistical Package for the Social Sciences)version 26.0. Normally distributed quantitative data are presented as the mean ± standard deviation and were compared between groups using an analysis of variance. Quantitative data that were not normally distributed are expressed as the median (interquartile interval) and were compared between groups using a nonparametric rank sum test. Qualitative data are presented as the percentage and were compared between groups using a chi-square test or a corrected chi-square test. For the primary outcome measures, binary meta-logistic regression was performed after adjustment for confounding factors. A P value of < 0.05 was considered statistically significant.

## Results

Eighty-nine patients who had undergone their first postoperative IVF/ICSI-assisted pregnancy cycle were included in this retrospective analysis. Of the 89 patients, 38, 27, and 24 were included in the PPOS, GnRH antagonist, and microstimulation groups, respectively. No significant differences were noted in the age of the female patients and their spouses and in the BMI and basal endocrine levels (FSH and oestradiol) of the female patients among the three groups (P > 0.05). The GnRH antagonist group had a significantly higher AFC than did the other two groups (P < 0.05; Table 1).

**Table 1:** The general data of patients in four groups

Item	Progestin-primed ovarian stimulation group	GnRH-antagonist group	Microstimulation group	Z/x2 value	P value
No. of cases	38	27	24		
Female age(year)	33.34 ± 4.14	32.30 ± 3.83	34.13 ± 4.50	1.331	0.270
Male age (year)	35.21 ± 7.88	33.11 ± 3.11	34.67 ± 4.92	0.990	0.376
Female body mass index( kg/m^2^)	22.93 ± 2.81	23.76 ± 3.11	23.81 ± 2.92	0.920	0.402
Duration of infertility (year)	4.01 ± 3.58	4.41 ± 3.15	2.99 ± 2.87	1.226	0.299
Antral follicle count	6.16 ± 2.46	11.30 ± 6.72^bc^	6.50 ± 3.43	12.008	P < 0.001
Basal follicle stimulating hormone (IU/L)	9.69 ± 5.02	8.20 ± 3.02	10.23 ± 4.35	1.565	0.215
Basal luteinizing hormone (IU/L)	12.91 ± 42.90	4.73 ± 2.09	4.55 ± 2.28	0.936	0.396
Main etiology of infertility (%)					
Tubal factor	52.6 (20/38)	44.4 (12/27)	70.8 (17/24)	4.623	0.099
Diminished ovarian reserve	73.7 (28/38)	66.7 (18/27)	54.1 (13/24)	1.913	0.384
Male factor	26.3 (10/38)	22.2 (6/27)	16.1 (4/24)	0.655	0.721
Other factors	57.8 (22/38)	62.9 (17/27)	70.8 (17/24)	1.596	0.450
Starting dosage of Gn	286.32 ± 72.12^a^	230.56 ± 65.63	265.63 ± 49.35	4.088	0.020
Dosage of Gn used	2806.58 ± 747.25	2467.11 ± 755.83	2818.75 ± 742.44	2.013	0.140
Endometrial thickness on HCG injection day	8.03 ± 1.74	10.50 ± 2.29^bc^	6.93 ± 1.83	23.053	P < 0.001

^a^Comparison with GnRH-antagonist group

^b^Comparison with microstimulation group

^c^Comparison with PPOS group

The starting dosage of Gn significantly differed among the three groups (P < 0.05). The GnRH antagonist group was administered a significantly lower starting dosage of Gn than the PPOS group. However, the total dosage of Gn did not significantly differ among the three groups. The endometrial thickness on the hCG injection day in the GnRH antagonist group was significantly higher than that in the other two groups (Table 1).

The cumulative pregnancy rate, cumulative live birth rate, viable embryo rate, and blastocyst formation rate did not significantly differ among the three groups (P > 0.05). The numbers of oocytes retrieved and 2PN embryos in the GnRH antagonist group were superior to those in the other two groups. However, the oocyte output rate was significantly higher in the microstimulation group than in the other two groups. The rate of high-quality embryos significantly differed among the three groups, with the PPOS group exhibiting the highest rate, followed by the GnRH antagonist and microstimulation groups (54.9% [67/122] vs. 46.6% [62/133] vs. 29.7% [19/64], x2 = 10.749, P < 0.005; Table 2).

**Table 2:** Comparison of clinical outcomes of patients among three protocols

Item	Progestin-primed ovarian stimulation group	GnRH-antagonist group	Microstimulation group	Z/x2 value	P value
No. of oocytes retrieved	4.31 ± 2.01	7.42 ± 5.17^bc^	3.88 ± 2.31	8.512	P < 0.001
Oocytes output rate (%)	63.2 (96/152)	57.9 (136/235)	91.2(52/57)^ac^	22.208	P < 0.001
No.2PN	3.39 ± 1.69	5.11 ± 4.09^bc^	2.67 ± 2.06	5.595	0.005
Available embryo rate (%)	83.6 (102/122)	90.2 (120/133)	90.9 (50/64)	3.216	0.200
High-quality embryo rate (%)	54.9 (67/122)	46.6(62/133)^bc^	29.7(19/64)^c^	10.749	0.005
Blastocyst formation rate (%)	65.2 (30/46)	57.8 (48/83)	50.0 (11/22)	1.518	0.468
Clinical pregnancy rate (%)	44.7 (17/38)	55.6 (15/27)	29.2 (7/24)	3.617	0.164
Cumulative live birth rate (%)	34.2(13/38)	48.1 (13/27)	20.8 (4/24)	4.188	0.123

^a^Comparison with GnRH-antagonist group

^b^Comparison with microstimulation group

^c^Comparison with PPOS group

Binary logistic regression analysis of the cumulative live birth rate was performed using the cumulative live birth rate as the observation index. This analysis controlled for confounding factors, namely the female patient’s age (continuous variable), the female patient’s BMI (continuous variable), infertility type (primary/secondary), AFC (continuous variable), and ovulation stimulation protocol (GnRH antagonist, PPOS, and microstimulation protocols). The cumulative live birth rate in the microstimulation group was lower than that in the GnRH antagonist group (adjusted odds ratio [aOR] = 0.153, 95% confidence interval [CI] 0.036–0.654, P = 0.011). However, the cumulative live birth rate did not significantly differ between the PPOS and GnRH antagonist groups (aOR = 0.332, 95% CI 0.093–1.18, P = 0.088; Table 3).

**Table 3:** Binary logistic regression analysis of factors affecting cumulative live birth rate

Factors	Wald	aOR	95% CI	P值
Maternal age	0.250	1.037	0.891–1.198	0.617
Female body mass index	1.176	0.908	0.763–1.081	0.278
Type of infertility (primary/secondary)	0.166	1.265	0.409–3.908	0.683
Antral follicle count	0.573	0.958	0.858–1.070	0.449
COH protocols				
GnRH-antagonist group		1		
Progestin-primed ovarian stimulation group	2.906	0.332	0.093–1.180	0.088
Microstimulation group	6.428	0.153	0.036–0.654	0.011

## Discussion

Endometriosis is a common gynaecological condition in women of childbearing age. This condition can distort the anatomical structures of the fallopian tubes and ovaries [8], leading to inflammation [9, 10], oxidative damage [11], and harm to oocytes [12], and harm to oocytes [13]. Currently, laparoscopic enucleation is the preferred treatment modality for ovarian cysts measuring ≥ 3 cm in diameter [14]. However, studies have indicated that women who undergo this surgery may respond poorly to Gn stimulation, produce fewer oocytes, and experience DOR, resulting in higher cycle cancellation rates of IVF and embryo transfer cycles as well as lower embryo implantation and clinical pregnancy rates [15]. Controlled ovulation induction is a crucial step in assisted reproductive technology (ART), and a personalised approach can substantially improve the clinical outcomes of ART. A previous study reported that growth hormone or percutaneous androgen supplementation during ovulation induction therapy significantly improved the cumulative pregnancy rate and live birth rate in patients with a poor ovarian response [16]. Currently, ovulation induction protocols commonly used in clinical practice for patients with DOR include GnRH antagonist, microstimulation, and PPOS protocols. For patients experiencing DOR following laparoscopic cystectomy of ovarial endometrioma, determining how to obtain high-quality oocytes and embryos through appropriate ovulation induction protocols to reduce stress among patients is an urgent problem in clinical practice.

Most studies have indicated that patients with endometriosis should undergo an ultra-long treatment regimen, mainly due to the inhibitory effect of the GnRH agonist on the ectopic endometrial tissue [17]. However, a review published in 2021 suggested that the GnRH antagonist or PPOS protocol may be more suitable than ultra-long treatment regimens for patients with endometriosis [18]. GnRH antagonists can competitively bind to pituitary GnRH receptors, reducing the secretion of pituitary Gn. Because of their direct antagonist action [19], GnRH antagonists maintain pituitary responsiveness and can thus maximise the ovarian response to Gn.

A retrospective study conducted in China in 2019 [20] compared the assisted reproductive outcomes of patients with endometriosis using GnRH antagonists, long agonist protocol and prolonged agonist protocol. The results revealed no significant differences in hCG positivity, clinical pregnancy, and total embryo implantation rates among the three groups. However, among patients with DOR, the GnRH antagonist group had a higher viable embryo rate than did the rectangular regimen group, and both the total numbers of Gn administration and medication days were lower in the GnRH antagonist group than in the other two groups. The use of GnRH antagonists in patients with endometriosis and infertility resulted in similar pregnancy outcomes as the rectangular and ultra-long regimens. However, GnRH antagonists reduced treatment costs and time and resulted in a higher viable embryo rate. A meta-analysis [21] published in 2022 included 13,050 cycles, with 5984 patients using a GnRH antagonist and 7066 patients not using an antagonist. The results demonstrated that the use of a GnRH antagonist was associated with higher live birth rates. Moreover, a significant improvement in live birth rates was noted in women with an AMH level of < 1 ng/mL and women aged ≥ 35 years. The findings indicate that the use of GnRH antagonists significantly reduces the cycle cancellation rate and increases the number of frozen embryos, possibly due to improvements in embryo quality and endometrial receptivity. This finding is consistent with the results of the present study, which demonstrated that the GnRH antagonist group had a higher rate of high-quality embryos than did the microstimulation group.

In 2015, Yanping [22] first proposed the PPOS protocol, which involves adding exogenous progesterone during the follicular phase. A meta-analysis published in 2021 [23] reported that in the DOR population, the PPOS protocol resulted in a lower incidence of early-onset luteinising hormone (LH) peaks and ovarian hyperstimulation syndrome than did the GnRH antagonist regimen, GnRH agonist regimen, and natural cycle.

The results of this study revealed no significant difference in the cumulative pregnancy rate among the three groups. However, the cumulative live birth rate was higher in the GnRH antagonist and PPOS groups than in the microstimulation group. This finding is similar to that of a retrospective study [24] conducted in China in 2020, which included 285 patients according to the Poseidon standard. The results showed that the cumulative clinical pregnancy rate was higher in the GnRH antagonist group than in the microstimulation and PPOS groups. Moreover, in the present study, the numbers of retrieved eggs and 2PN embryos in the GnRH antagonist group were higher than those in the other two groups. This finding may be associated with the larger dosage and longer duration of Gn treatment in the GnRH antagonist protocol, which provides more opportunities for follicular recruitment and growth. Thus, for patients with DOR following laparoscopic cystectomy of ovarial endometrioma, the GnRH antagonist protocol is a more suitable ovulation induction regimen than the microstimulation protocol.

In 2019, a domestic meta-analysis [25] included 2270 cycles in the PPOS group and 2463 cycles in the microstimulation group. The results revealed that for patients with DOR, the PPOS protocol resulted in a higher rate of high-quality embryos and a lower rate of cycle cancellation than did the microstimulation protocol. This finding is consistent with the results of the present study. The microstimulation protocol, which does not involve downregulation, is prone to premature LH peaks and follicular ovulation, resulting in a higher rate of cycle cancellation. However, the results of this study indicate that compared with the microstimulation protocol, the PPOS protocol led to a higher cumulative activity rate. Therefore, the PPOS protocol resulted in better clinical outcomes in the patients included in the current study.

The results of this study are inconsistent with those of a retrospective study [26] published in 2018, which included 186 POR patient cycles from 2014 to 2016. The results showed that the PPOS protocol resulted in a higher clinical pregnancy rate and live birth rate than did the GnRH antagonist protocol. The inconsistency in findings may be attributable to differences in the included population and sample size between the previous and current study.

Few studies have evaluated the effectiveness of ovulation induction protocols after laparoscopic cystectomy of ovarial endometrioma. This study included patients experiencing DOR after laparoscopic cystectomy of ovarial endometrioma and investigated the effectiveness of PPOS, microstimulation, and GnRH antagonist protocols by determining the cumulative live birth rate. A limitation of this study is the small sample size, which may have led to the lack of significant differences between the main observation indicators of this study. As of the data collection period, there were still many embryos that were frozen and had not been transferred or had not yet resulted in a live birth following successful pregnancy. Furthermore, some patients were still pregnant. Further follow-up is essential to obtain more informative results. In addition, ovarian cysts are highly complex in nature. More research is needed to determine the optimal ovulation induction plan for patients following ovarian cyst surgery.

## Conclusion

In conclusion, for patients aged under 40 years who experienced DOR after laparoscopic cystectomy of ovarial endometrioma, GnRH antagonist protocol and PPOS protocol can obtain better ovulation induction outcomes and cumulative live birth rate than microstimulation protocol, and are more suitable ovulation induction protocols. Due to the effect of PPOS protocol and microstimulation protocol on endometrial receptivity, it is necessary to undergo whole embryo freezing followed by frozen-thawed embryo transfer, but this study has not yet compared the time to achieve live birth and specific medical costs of these three ovarian stimulation protocols. In future, further research is needed to investigate the association between the three ovarian stimulation protocols and assisted pregnancy outcomes with lower economic costs.

## Data Availability

The data sets used and/or analyzed during the current study are available from the corresponding author on reasonable request.

## Abbreviations

Gn: Gonadotropin
GnRH: Gonadotropin releasing hormone
PPOS: Progestin-primed ovarian stimulation
DOR: Diminished ovarian reserve
IVF: In vitro fertilization
ICSI: Intracytoplasmic sperm injection
AMH: Anti-Mullerian hormone
AFC: Antral follicle count
FSH: Follicle-stimulating hormone
BMI: Body mass index
hCG: Human chorionic gonadotropin
FET: Frozen–thawed embryo transfer
2PN: Two pronuclear
LH: Luteinising hormone
